# INVESTIGATING THE CELLULAR AND MOLECULAR EFFECTS OF AGING ON FAT TISSUE IN DROSOPHILA MELANOGASTER

**DOI:** 10.1093/geroni/igad104.0328

**Published:** 2023-12-21

**Authors:** Justice Bell, Isaiah Williams, Tancia Bradshaw, Alissa Armstrong

**Affiliations:** Claflin University, Charlotte, North Carolina, United States; University of South Carolina, Columbia, South Carolina, United States; University of South Carolina, Columbia, South Carolina, United States; University of South Carolina, Columbia, South Carolina, United States

## Abstract

Adipose tissue, also known as fat tissue, is an energy reservoir responsible for metabolic homeostasis. Changes in adipocytes during aging have been associated with obesity, insulin resistance, type two diabetes, and cancer. Thus, in response to the obesity epidemic and the United States aging population, the goal of this experiment is to understand the cellular and molecular effects of aging on adipocytes. In doing so, drosophila were aged at 5,10,15, and 20 days and dissected for adipose tissue. We analyzed adipocyte size, lipid droplet composition, and immune response pathway activity. In this experiment we used the immunostaining process to visualize adipocytes across all ages. Cellular and nuclear size were quantified using ImageJ software. Immune response pathways were examined using reverse transcriptase polymerase chain reaction and gel electrophoresis. Expression of antimicrobial peptides were measured and analyzed against a control, RP49. Our results revealed a positive correlation between fly age, adipocyte and nuclear size. Thus, we can associate aging with increased adipocyte size. Various trends in AMP expression were observed; we have contributed this to the variation in function of each AMP. Further directions include characterizing the effect of aging on AMP expression and examining the relationship between adipocyte size and AMP expression in aging.

